# Human papillomavirus prevalence, viral load and pre-cancerous lesions of the cervix in women initiating highly active antiretroviral therapy in South Africa: a cross-sectional study

**DOI:** 10.1186/1471-2407-9-275

**Published:** 2009-08-07

**Authors:** Jennifer R Moodley, Deborah Constant, Margaret Hoffman, Anna Salimo, Bruce Allan, Ed Rybicki, Inga Hitzeroth, Anna-Lise Williamson

**Affiliations:** 1 School of Public Health and Family Medicine, Women's Health Research Unit, University of Cape Town, Cape Town, South Africa; 2 Institute of Infectious Disease and Molecular Medicine, University of Cape Town, Cape Town, South Africa; 3 Department of Molecular and Cell Biology, University of Cape Town, Cape Town, South Africa; 4 National Health Laboratory Service, Groote Schuur Hospital, Cape Town, South Africa

## Abstract

**Background:**

Cervical cancer and infection with human immunodeficiency virus (HIV) are both important public health problems in South Africa (SA). The aim of this study was to determine the prevalence of cervical squamous intraepithelial lesions (SILs), high-risk human papillomavirus (HR-HPV), HPV viral load and HPV genotypes in HIV positive women initiating anti-retroviral (ARV) therapy.

**Methods:**

A cross-sectional survey was conducted at an anti-retroviral (ARV) treatment clinic in Cape Town, SA in 2007. Cervical specimens were taken for cytological analysis and HPV testing. The Digene Hybrid Capture 2 (HC2) test was used to detect HR-HPV. Relative light units (RLU) were used as a measure of HPV viral load. HPV types were determined using the Roche Linear Array HPV Genotyping test. Crude associations with abnormal cytology were tested and multiple logistic regression was used to determine independent risk factors for abnormal cytology.

**Results:**

The median age of the 109 participants was 31 years, the median CD4 count was 125/mm^3^, 66.3% had an abnormal Pap smear, the HR-HPV prevalence was 78.9% (Digene), the median HPV viral load was 181.1 RLU (HC2 positive samples only) and 78.4% had multiple genotypes. Among women with abnormal smears the most prevalent HR-HPV types were HPV types 16, 58 and 51, all with a prevalence of 28.5%. On univariate analysis HR-HPV, multiple HPV types and HPV viral load were significantly associated with the presence of low and high-grade SILs (LSIL/HSIL). The multivariate logistic regression showed that HPV viral load was associated with an increased odds of LSIL/HSIL, odds ratio of 10.7 (95% CI 2.0 – 57.7) for those that were HC2 positive and had a viral load of ≤ 181.1 RLU (the median HPV viral load), and 33.8 (95% CI 6.4 – 178.9) for those that were HC2 positive with a HPV viral load > 181.1 RLU.

**Conclusion:**

Women initiating ARVs have a high prevalence of abnormal Pap smears and HR-HPV. Our results underscore the need for locally relevant, rigorous screening protocols for the increasing numbers of women accessing ARV therapy so that the benefits of ARVs are not partially offset by an excess risk in cervical cancer.

## Background

Cervical cancer and infection with human immunodeficiency virus (HIV) are both important public health problems in South Africa (SA). Among South African women cervical cancer is the second commonest cancer, with an age standardized incidence rate of 30 per 100 000 per year, and is the leading cause of cancer mortality [1]. SA is confronted by one of the worst HIV epidemics in the world and it is estimated that there are currently 5.4 million people with HIV/AIDS in SA, with women more severely affected than men [2]. Studies have shown that women infected with HIV have a higher prevalence of human papillomavirus (HPV) infection, are more likely to develop persistent HPV infection, are more frequently infected with multiple HPV types and are at a greater risk of developing cervical squamous intraepithelial lesions (SIL) and cervical cancer. These lesions are more aggressive, persistent and more likely to recur following treatment [3-11]. A recent study in South Africa has confirmed that HIV positive women are at an increased risk of cervical SILs [12].

Highly active antiretroviral therapy (HAART) has been shown to decrease HIV viral loads, increase CD4 cell counts and decrease most opportunistic infections. Since the introduction of HAART there has been a decline in certain malignancies in HIV infected individuals [13,14]. However studies on the impact of HAART on the natural history of cervical squamous intraepithelial lesions (SILs) have produced inconsistent results [15,16]. As anti-retroviral (ARV) therapy becomes increasingly available in the public sector in South Africa, the life expectancy of HIV positive women will increase. HPV-associated disease will also become more clinically relevant and rigorous screening protocols are needed so that the benefits of ARVs are not partially offset by an excess risk in cervical cancer. These screening protocols need to be locally relevant. Few studies have examined the prevalence of cervical dysplasia in women initiating ARVs in South Africa [17]. The aim of this study was to determine the prevalence of cervical SILs, high-risk HPV (HR-HPV), HPV viral load and HPV genotypes in HIV positive women initiating ARV therapy.

## Methods

A cross sectional survey was conducted at a public sector ARV treatment clinic in Cape Town, South Africa, between January and May 2007. Women, eighteen years and older, who were being considered for initiation of ARV therapy were informed of the study by the clinic staff. Those indicating interest were referred to the research nurse, who explained the details of the study and obtained informed consent. Women who had a hysterectomy, were menstruating or pregnant at the time of the study were excluded. The research nurse collected data on socio-demographic status, sexual behavior, history of a sexually transmitted infection (STI), obstetric and gynaecology history and risk factors for cervical cancer using a structured questionnaire; conducted a pelvic examination; took a Pap smear using a cytobrush and collected cervical samples for HPV testing with Digene cervical samplers. The samples for HPV testing were stored in Digene transport medium at -80°C until required. CD4 count data were extracted from clinical records. Pap smears were interpreted at the South African National Health Laboratory Service and classified according to the Bethesda classification system [18]. Women with high-grade squamous intraepithelial lesions (HSIL) were referred to the regional colposcopy clinic and those with other abnormalities asked to return for repeat Pap smears according to the clinic protocol. Cervical samples were assayed for HPV infection using the Digene Hybrid Capture 2 (HC2) assay which detects13 HR-HPV genotypes. The HC2 results are given as a relative light unit (RLU) ratio which is the ratio of light emitted by the specimen to the light emitted from the mean RLU of triplicate positive control specimens containing 1 pg/ml of HPV DNA (5000 copies of HPV genome). Specimen RLU values were converted into a ratio to the cut-off value (1 pg/ml). Specimens with a ratio of < 1.00 were considered negative, and those with a ratio of ≥ 1.00 were considered positive. Low, but positive RLU values were not retested and were considered positive for the purposes of the analysis. HPV types were determined using the Roche Linear Array HPV Genotyping test, which identifies 37 different HPV genotypes. The cyto-technician and laboratory technician were blinded to the clinical profile of the clients.

Data analysis was conducted using the statistical program STATA 9.0 (STATA Corporation, College Station, Texas). Descriptive statistics (medians and proportions) were used to characterize the variables. Basic crude associations with abnormal cytology were tested using Wilcoxon Rank Sum tests for medians and Chi-square and Fischer's Exact tests for proportions. Multivariate logistic regression was carried out to determine independent risk factors for low-grade squamous intra-epithelial lesions (LSILs) and high-grade squamous intra-epithelial lesions (HSIL). To avoid co-linearity in the model between HPV infection measured by the HC2 test and HPV viral load, we derived categories of HPV infection as follows: HPV HC2 negative as the referent group and HPV HC2 positive in categories according to the median viral load. Other variables included in the model are known to be associated with cervical abnormality and were: age, number of sexual partners, age at first sexual intercourse and CD4 count. Median values were used as cut-offs to categorize these variables. Ethical approval was granted by the Research Ethics Committee of the University of Cape Town.

## Results

A total of 120 women were referred by clinic staff to participate in the study. Nine women were excluded because they were menstruating and two declined to participate because of time constraints. In total 109 women were included in the study. The socio-demographic and reproductive characteristics for the overall study population and according to cervical cytology results are presented in Table 1. A total of 98/109 women had an adequate Pap smear and of these 65 (66.3%) had an abnormal Pap smear. Atypical squamous cells of undetermined significance (ASCUS) was reported in 15 (15.3%), LSIL in 39 (40.0%), HSIL in 10 (10.2%) and atypical squamous cells-cannot exclude HSIL (ASC-H) in 1 (1.0%) of women. For analysis the cytology results were grouped as normal/ASCUS versus LSIL/HSIL.

**Table 1:** Socio-demographic and reproductive characteristics for the overall study population and according to cervical cytology results

Characteristic	Overall N = 109	Normal/ ASCUS Pap smear N = 48	LSIL/HSIL Pap smear N = 49	P value^a^
		
	Data are N (%) except where otherwise specified			
Median age in years (range)	31 (20 – 61)	32 (20 – 57)	30 (20 – 61)	0.43

Employed	21 (19.3)	12 (25)	7 (14.3)	0.18

Ever smoked	21 (19.3)	5 (10.4)	16 (32.7)	0.03

Married or in stable relationship	52 (47.7)	22 (45.8)	23 (46.9)	1.00

Median years of school attendance (range)	10 (0 – 13)	10 (0 – 13)	9 (0 – 13)	0.42

Ever pregnant	89 (81.7)	40 (83.3)	39 (79.6)	0.64

Ever used injectable contraception	97 (89.0)	43 (89.6)	42 (85.7)	0.56

Ever used oral contraception	29 (26.6)	11 (22.9)	13 (26.5)	0.68

Ever used condoms	55 (50.5)	21 (43.8)	29 (59.2)	0.13

Median age of first intercourse (range)	17 (4 – 25)	17 (13 – 21)	16 (4 – 25)	0.12

Median number of sexual partners (range)	4 (1 – 13)	4 (1 – 10)	4 (1 – 13)	0.15

Ever had an STI	92 (84.4)	41 (85.4)	42 (85.7)	0.97

Past Pap smear	46 (42.2)	23 (47.9)	16 (32.7)	0.10

Median CD4 count (range)	125 (14 – 1047)	125.5 (14 – 1047)	123 (32 – 313)	0.95

HR-HPV present (Digene)	86 (78.9)	28 (58.3)	47 (95.9)	< 0.001

HR-HPV present (Roche)	97 (89.0)	37 (77.1)	49 (100)	< 0.001

Median HPV viral load in RLU (range) All samples	64.3 (0.12 – 2248.95)	3.19 (0.12–1373.77)	365.49 (0.21–2248.95)	< 0.001

Median HPV viral load in RLU (range) Only HC2 positives	181.06 (1.19–2248.95)	60.3 (1.19–1373.77)	444.07 (11.98–2248.95)	0.001

Prevalence of multiple HPV types Only HC2 positives	76 (78.4)	23 (62.2)	45 (91.8)	0.001

^a ^P values are from Wilcoxon Rank Sum tests for medians and chi-squared tests for proportions or Fisher's exact tests where numbers in the cells were < 5.

ASCUS, atypical squamous cells of undetermined significance; LSIL, low grade squamous intra-epithelial lesions; HSIL, high-grade squamous intraepithelial lesions; HPV, human papillomavirus; STI, sexually transmitted infection; HR-HPV, high-risk human papillomavirus; RLU, relative light units; HC2, Hybrid Capture 2

Overall the median age was 31 years (range 20 – 60 years). The majority of women were unemployed (80.7%), just under half (47.7%) were either married or in a stable relationship and most participants had attended high school (median of 10 years of schooling). There were no significant socio-demographic differences between women with normal/ASCUS Pap smears and women with LSIL/HSIL Pap smears.

The majority of the participants (55.0%) were currently on contraception (data not shown). Of these, 40% were using condoms only, 28% condoms and another contraceptive method, 23% injectable contraception, 2% oral contraception and 6% another method. There was no significant difference in contraceptive use between women with a normal/ASCUS Pap smear and those with a LSIL/HSIl Pap smear. Most women reported having had an STI (84.4%). Of these 27.1% gave a history of having an STI in the preceding week. The median CD4 cell count was 125.0/mm^3 ^(range 14.0 – 1047.0/mm^3^), with no significant difference in the median CD4 count between women with normal/ASCUS Pap smears and women with LSIL/HSIL Pap smears.

Women with LSIL/HSIL Pap smears had a significantly higher prevalence of HR-HPV compared to those with normal/ASCUS Pap smears on both the Digene (95.9% vs. 58.3% and Roche assays (100% vs. 77.1%), had a higher median HPV viral load (444.1 vs. 60.3 RLU) among those with HC2 positive samples and a higher prevalence of multiple HPV types present (91.8% vs. 62.2%). Of the 109 participants: 85 tested positive on both the Roche Linear Array and Digene HC2 tests, 12 were positive on the Roche but negative on the Digene test, 11 were negative on both tests and 1 was negative on the Roche test but positive on the Digene test. The prevalence of HR-HPV using the Digene test was 78.9% (95% CI 69.8% – 85.9%) and using the Roche Linear Array HPV Genotyping test the prevalence of HR-HPV was 89.0% (95% CI 81.20 – 93.93).

The estimated median HPV viral load was 64.8 RLU for all samples that had HC2 testing and 181.1 RLU for those that samples that were HC2 positive based on the 1.0 pg/ml clinical cut-off point. Table 2 provides information on the median HC2 viral load and inter-quartile range by each category of cervical abnormality. There was a significant trend of increasing HPV viral load with increasing severity of cytological abnormality both when restricting the analysis to HC2 positives (p = 0.002) and when including all samples (p < 0.0001). Both analyses showed an overlap in the range of viral loads for the cytology categories.

**Table 2:** HPV viral load and cervical cytology

Cervical cytology	N	Median HPV Viral load in RLU^a^ (interquartile range)
Normal	33	1.19 (0.19 – 14.30)
	*17*	*14.3 (3.28 – 190.06)*

ASCUS	15	48.19 (0.59 – 218.51)
	*11*	*90.27 (33.59 – 232.08)*

LSIL	39	308.33 (54.31 – 1259.64)
	*37*	*365.49 (82.06 – 1259.64)*

HSIL	10	594.55 (177.5 – 1504.88)
	*10*	*594.55 (177.5 – 1504.88)*

For HC2 all samples: P_trend _< 0.0001		
*For HC2 positives only: P_trend _= 0.002*		

^a^Viral load in regular types represents viral load from all samples with HC2 testing and in italics from the HC2 positive samples only

HPV, human papillomavirus; HC2, Hybrid Capture 2; RLU, relative light units; ASCUS, atypical squamous cells of undetermined significance; LSIL, low grade squamous intra-epithelial lesions; HSIL, high-grade squamous intraepithelial lesions

When stratified by CD4 count category, median HPV viral loads were as follows: for those with CD4 < 100 (N = 38), median HPV viral load = 23.5 RLU; CD4 ≥100 < 200 (N = 63), median HPV viral load = 97.3 RLU and CD4 ≥200 (N = 8), HPV viral load = 8.9 RLU. The correlation between log CD4 load and log HPV viral load was not significant (correlation coefficient r = -0.005)

**Figure 1:**
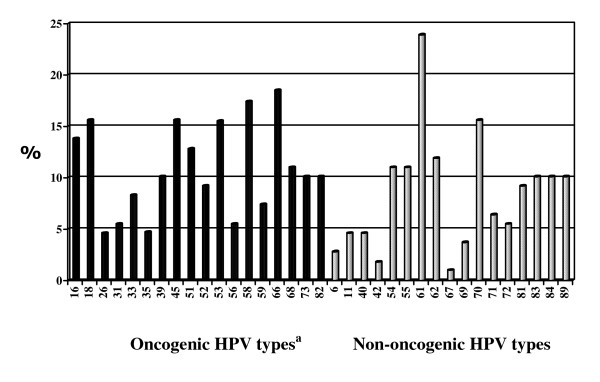
**Prevalence of HPV types detected in 109 women initiating HAART**. ^a^Types 26, 53 and 66 are usually classified as probably oncogenic, but in this analysis were grouped with oncogenic types to simplify interpretation.

On Roche Liner Array testing a total of 35 HPV genotypes were detected in the 109 women. The median number of HPV types per woman was 3 (range 0 – 12), with multiple genotypes (≥2 types) being detected in 69.7% of all participants and in 78.4% of participants with a positive HC2 test. Figure 1 gives the prevalence of HPV types detected. Overall HPV types 61 and 66 were most commonly detected, with a prevalence of 23.9% and 18.5% respectively. The prevalence of HPV 16 and 18 was 13.8% and 15.6% respectively and the prevalence of types 6 and 11 was 2.75% and 4.6%, respectively. Among women with abnormal smears the most prevalent HR-HPV types were HPV types 16, 58 and 51, all with a prevalence of 28.5%, followed by HPV types 66 (24.6%), 18 (21.5%) and 45 (20.0%). Among the 10 women with HSIL the most common HR-HPV type was HPV 45 (prevalence 40%), followed by HPV types 16, 35, 39, 58 and 51 all with a prevalence of 30% and HPV types 18, 31, 33 and 66 all with a prevalence of 20%.

Multivariate logistic regression was carried out to determine independent risk factors for HSIL/LSIL. The model showed that HPV viral load was associated with an increased odds of LSIL/HSIL. The odds of LSIL/HSIL was 10.7 (95% CI 2.0 – 57.7) for those that were HC2 positive and had a viral load of ≤ 181.1 RLU (the median HPV viral load), and for those that were HC2 positive with a HPV viral load > 181.1 RLU the odds was 33.8 (95% CI 6.4 – 178.9). The odds ratio (OR) for LSIL/HSIL for the other variables were as follows: age OR = 0.94 (95% CI 0.36 – 2.45), number of sexual partners OR = 0.88 (95% CI 0.31 – 2.47, age at first sexual intercourse OR = 0.47 (95% CI 0.16 – 1.32), and CD4 count OR = 1.11 (95% CI 0.42 – 2.96).

## Discussion

Our study of women initiating ARV therapy recorded an exceptionally high prevalence of cervical abnormalities (66%). This is much higher than that recorded in studies conducted among HIV positive women in developed countries [4,10,19-21] and is one of the highest recorded in Africa [9,12,22,23]. Previous studies conducted among HIV positive women in the Western Cape Province in South Africa have recorded abnormal Pap smear prevalence of 50 to 55% [12,17]. These latter studies were conducted in women with either an unknown CD4 status or a higher median CD4 status than was recorded in our study. The associations between low CD4 counts, HPV and cervical dysplasia have been previously documented [10,20]. Given the low CD4 counts in our study population, severe immunosuppression is the most likely explanation for the high prevalence of cervical abnormalities and HPV detected. The only study to report a higher prevalence of cytological abnormalities than our study, was that conducted by Parham et al among women attending an HIV-care clinic at a tertiary centre in Zambia (abnormal Pap smear prevalence of 94%) [22]. The latter study was conducted among women with a median CD4 count of < 200 μ/L and of a similar age to our study population. However in the Parham study cytological abnormalities were detected by monolayer liquid cytology, which is known to have a higher sensitivity than conventional cytology, used in our study. The high prevalence of cervical abnormalities, in this sub-group of severely immuno-compromised HIV positive women, has major health planning and resource implications, particularly as ARVs become increasingly more available in South Africa. The results underscore the importance of developing screening and management guidelines for women initiating ARVs that take into account the enormous disease burden in the context of a constrained resource setting.

HR-HPV was present in 79% (Digene) of the women in our study. This is amongst the highest HR-HPV prevalence recorded in the literature [9,19,24] and is a cause for concern. Previous studies have documented an increase in HPV viral load with increasing severity of cervical lesions [25-27]. In this study we assessed HPV viral load using the HC2 test, which provides a semi-quantitative assessment of viral load. HC2 as a measure of viral load can be meaningful in some settings, however as this is a semi-quantitative test results need to be interpreted with caution, understood within the context of the setting and consideration given to the presence of single or multiple-type HPV infections. Some studies have shown that HC2 results can be considered as reflective of HPV viral load [28] while others have not [29]. It remains controversial if the HC2 test will give meaningful results in cervical specimens from HIV positive women who are more likely to be infected with multiple HPV types compared to HIV negative women [30]. HIV positive women are at higher risk of cervical abnormalities but it is not known how many HPV types, in HIV positive women infected with multiple HPV types, contribute to cervical disease [26]. In this paper we investigated whether an increased HPV viral load, as measured by HC2, reflects increased risk of cervical disease if the result is a combination of many HPV types instead of only one type and found an association between HPV viral load and severity of cervical cytological abnormality. We speculate that HPV viral load might be clinically useful in predicting cervical dysplasia in HIV positive women if a higher cut-off point is used. However any change in the cut-off point will result in changes in test sensitivity as well and longitudinal studies are needed to test this hypothesis. It has been previously reported that cervical intraepithelial neoplasia with high viral loads are more likely to persist than those with a low level of HR-HPV [31]. It is critical that we determine the natural history of HR-HPV among women initiating ARVs as it is known that women with persistent HR-HPV are at an increased risk of developing cervical abnormalities.

Clifford et al have shown that there is considerable variation in regional HPV type distribution [30]. In our study the most common prevalent HPV types among all women were types 61 (23.9%), 66 (18.5%), 58 (17.4%), 18 (15.6%), 45 (15.6%) and 70 (15.6%); and the most common types among women with cervical abnormalities were types16 (28.5%), 58(28.5%), 53(28.5%), 51(28.5%), 66 (24.6%), 18 (21.5%) and 45 (20.0%). As has been shown in other studies, HPV 16 did not predominate over other types to the same extent as is usually seen in HIV negative women [32-36]. The majority of our participants had multiple HPV genotypes. Although types 16 and 18 were among the commonest HPV types seen in women with cervical abnormalities, we found a relatively high prevalence of HPV types not covered by the HPV vaccine. This has been documented by others and has important implications for the potential impact of HPV vaccines currently available.

The small sample size limited our power to assess the association between LSIL/HSIL and potential predictors. A strong association between HIV viral load and cervical abnormalities has been demonstrated in other studies [10,20,37]. We were unable to collect data on HIV viral load. Another limitation was that cytological abnormalities could not be histologically confirmed in all women. However there is no reason to believe that the cytologists would have over-diagnosed cytological abnormalities because of the known association between HIV and cervical pre-cancerous lesions, as the cytologists were unaware of the participant's HIV status. It is possible that the true prevalence of cervical abnormalities might in fact be higher than we recorded, as the sensitivity of Pap smears has been shown to be as low as 20% [38,39].

## Conclusion

In summary, women initiating HAART in SA have an extremely high prevalence of abnormal Pap smears and HR-HPV and this has major health planning and resource implications. It is critical that we develop locally relevant, rigorous screening protocols for the increasing numbers of women accessing ARV therapy. Further studies are needed to explore the predictive role of HPV viral load using higher cut-off points in HIV-positive women.

## Competing interests

The authors declare that they have no competing interests.

## Authors' contributions

JM was involved in designing the study and in the data analysis, and drafted the manuscript. DC was involved in proposal writing and responsible for detailed data analysis. MH was involved in proposal writing, supervision of field staff and data analysis. AS carried out the HPV typing assessments and was involved in aspects of the data analysis. BA carried out the viral load assessments and was involved in data analysis. ER was involved in HPV typing assessments and aspects of data analysis. IH was involved in HPV typing assessments and aspects of data analysis. AW was involved in proposal writing, was responsible for virological assessments and involved in aspects of data analysis. All authors and read and approved the final manuscript.

## Pre-publication history

The pre-publication history for this paper can be accessed here:

http://www.biomedcentral.com/1471-2407/9/275/prepub
